# Bile acids: potential links to overweight/obesity and androgen levels in pubertal girls

**DOI:** 10.3389/fendo.2025.1695465

**Published:** 2025-11-11

**Authors:** Sofia Malave-Ortiz, Samantha A. M. McNeley, Sheri Denslow, Fred B. Lih, Natalie D. Shaw

**Affiliations:** 1 Pediatric Neuroendocrinology Group, Clinical and Translational Research Branch, National Institute of Environmental Health Sciences, National Institutes of Health (NIEHS/NIH), Durham, NC, United States; 2 DLH, LLC, Bethesda, MD, United States; 3 NIEHS Mass Spectrometry Research Center, National Institute of Environmental Health Sciences, National Institutes of Health (NIEHS/NIH), Durham, NC, United States

**Keywords:** bile acids, puberty, obesity, androgens, adolescent girls

## Abstract

**Background:**

Pubertal girls with higher body mass index (BMI) or total body fat (TBF) have higher androgens. We demonstrated that several bile acids (BAs) were associated with BMI, TBF, and androstenedione in an untargeted metabolomics study.

**Objective:**

To investigate the relationship between body composition, BAs, and androgens in pubertal girls.

**Methods:**

Blood samples were collected at up to seven study visits that included Tanner staging, breast ultrasound, and dual-energy x-ray absorptiometry. Serum total testosterone, free testosterone (FT), androstenedione, dehydroepiandrosterone sulfate, and 18 BAs were measured by liquid chromatography mass spectrometry. Generalized estimating equations estimated associations between TBF percent or BMI z-score, hormones, and BAs adjusted for time since enrollment, age, menarche status, race, and breast morphological stage. Exposures were taken from the preceding study visit (lagged).

**Results:**

Eighty-two participants (aged 10.9 ± 1.4 SD years; 55% non-Hispanic White, 29% non-Hispanic Black, 11% Hispanic, 6% Other; 65% normal weight, 35% overweight/obese) contributed an average of 2.59 samples. BAs were stable over time and not associated with menarchal status. BMI and TBF were negatively associated with total BAs (p_FDR_ = 0.0001). FT was nominally positively associated with two primary, conjugated BAs: taurocholic acid (*p* = 0.047) and taurodeoxycholic acid (*p* = 0.036).

**Conclusion:**

BAs are important signaling molecules with roles in metabolic and endocrine function. BMI and TBF were inversely associated with BAs, and two BAs were nominally positively associated with FT in girls across a spectrum of body weights. These results suggest novel biological links between altered BA signaling, overweight/obesity, and androgen production among pubertal girls.

## Introduction

In conducting the Body Weight and Puberty Study (BWPS), a 4-year longitudinal study of healthy pubertal, pre-menarchal girls in the Triangle region of North Carolina, we recently observed that girls with higher total body fat (TBF), as determined by dual x-ray absorptiometry (DXA), developed higher levels of total testosterone (TT) and free testosterone (FT) and androstenedione (AD) than girls with lower body fat in mid- to late puberty (1). These data are consistent with cross-sectional studies demonstrating an association between higher body mass index (BMI) and higher serum androgen levels in peri-pubertal and pubertal girls (2–7), including a notable recent study by Kim et al. that included a large, nationally representative sample of U.S. girls (8).

Insulin resistance in girls with overweight/obesity has been proposed to mediate their relative hyperandrogenism (4). However, we recently conducted an exploratory, untargeted metabolomics study in serum collected from BWPS participants that suggested a potential role for bile acids (BAs): we identified nominally significant associations between BAs and (1) BMI and TBF (inverse relationship) and (2) AD (positive relationship) (9).

**Figure 1:**
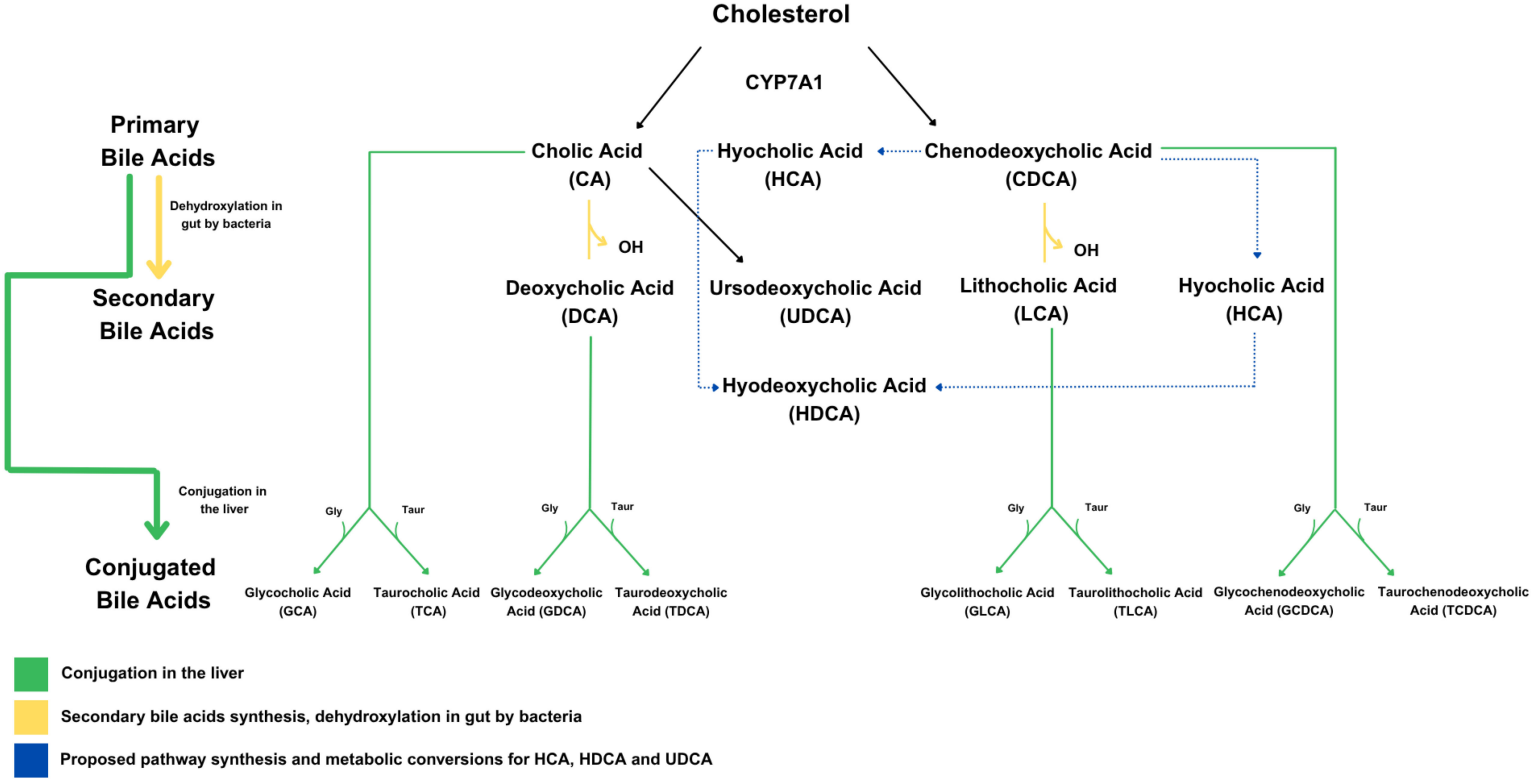
Bile acid synthesis pathways, including both classical and alternative routes. The classical BA pathway is the main route by which the liver converts cholesterol into BAs, starting with the enzyme CYP7A1. This process produces the two primary BAs—cholic acid (CA) and chenodeoxycholic acid (CDCA). Once secreted into the intestine, these two BAs can be modified by gut bacteria into secondary BAs such as deoxycholic acid (DCA) and lithocholic acid (LCA). In addition to this pathway, the synthesis of BAs such as ursodeoxycholic acid (UDCA), hyocholic acid (HCA), and hyodeoxycholic acid (HDCA) is thought to occur through an alternative route.

BAs are cholesterol derivatives synthesized in the liver by the rate-limiting enzyme cholesterol 7 alpha-hydroxylase (CYP7A1). The primary BAs, cholic acid (CA) and chenodeoxycholic acid (CDCA), are conjugated with either glycine or taurine in the liver, secreted into bile, and stored in the gallbladder until they are released into the small intestine in response to fatty or protein-rich foods. BAs are further modified into secondary BAs by colonic bacteria (**Figure 1**). BAs have recently been recognized as important signaling molecules with functions well beyond lipid digestion and absorption. By binding to two receptors, the nuclear receptor farnesoid X receptor (FXR) and the cell membrane G-protein coupled BA receptor (TGR5), they have been shown to influence a diverse set of biological systems, including metabolic, endocrine, and immune/inflammatory (10).

There has been very limited investigation into the relationship between body weight/composition or androgens and BAs in children. For example, while the composition of the BA pool was shown to be altered in one cross-sectional study of Italian children and adolescents with obesity (11), the majority of these children had obesity-related complications (e.g., hepatic steatosis, hypertension), limiting the generalizability of these findings. An additional study of Chinese youth whose main focus was on BAs in pediatric non-alcoholic fatty liver disease demonstrated that a subset of BAs were lower in children with overweight/obesity than in controls (12), and a small cross-sectional study of Canadian youth also demonstrated an inverse correlation between BAs and body fat percentage (13). The relationship between BAs and androgen levels has only been investigated in women with polycystic ovarian syndrome (PCOS) relative to regularly cycling adult controls (14–20), with mixed results.

To further explore the relationship between body weight/composition or androgens and BAs in healthy pubertal girls, we took advantage of our longitudinal BWPS cohort to assay a panel of clinically important BAs using mass spectrometry.

## Materials and methods

### Study participants

The characteristics of the BWPS participants have been reported previously (1, 21). All participants were healthy pre-menarchal girls with some breast development, per parental report, recruited from the community. They had no chronic medical conditions (including hepatic disease), were not taking any medications known to affect puberty, and did not have moderate or severe acne or hirsutism. The study was approved by the National Institute of Environmental Health Sciences (NIEHS) institutional review board. Signed informed assent and consent were obtained from each participant and her parent, respectively.

### Protocol

The participants who contributed samples for this Body Weight and Puberty sub-study completed one to seven study visits (mean ± SD: 2.59 ± 1.46 visits). All visits included anthropometrics (height, weight, waist-hip ratio), a physical exam with Tanner staging of the breast and pubic hair, breast ultrasound for breast morphological staging, non-fasting blood collection (optional after visit 1), and DXA (at the first two study visits) to determine percent body fat and the fat-free mass index [FFMI; fat-free mass (kg) divided by height (m^2^)], as previously described (1, 22). Parents were questioned regarding the participant’s onset of menarche at each study visit and through follow-up phone calls. All study procedures were conducted at the Clinical Research Unit of the National Institute of Environmental Health Sciences or off-site at a private medical imaging facility.

### Laboratory measurements

Serum TT, AD, and dehydroepiandrosterone sulfate (DHEAS) levels were measured by liquid chromatography–tandem mass spectrometry (LC-MS/MS; Triple Quad 6500 LC-MS/MS System, AB SCIEX) at the Division of Laboratory Sciences, National Center for Environmental Health, Centers for Disease Control and Prevention, as previously described (1). The assay limits of detection and limits of quantification are TT 0.57 ng/dl and 1.91 ng/dL; AD 0.82 ng/dl and 2.75 ng/dl; and DHEAS 0.22 mcg/dl and 2.83 mcg/dl, respectively. Sex hormone–binding globulin (SHBG) was measured using a chemiluminescent immunoassay (Siemens Immulite 2000 XPi analyzer). FT was calculated from TT, SHBG, and albumin (set at 4.3 g/dl) using the equation developed by Vermeulen et al. (23). Values below the limit of detection were imputed with one-half the minimum observed value. A panel of 18 BAs (including primary, secondary, conjugated, and unconjugated forms; **Figure 1**) was measured using LC-MS/MS at NIEHS. Liver function tests were not performed.

### Statistical analysis

BA and hormone values were natural log transformed and standardized. Body composition variables were analyzed as BMI Z-scores and TBF percent. Generalized estimating equations (GEE) with an autoregressive covariance structure were used to examine associations (1): between TBF or BMI, time since baseline, and menarchal status (exposures) and BAs (outcomes), and (2) between BAs (exposure) and hormone level (outcome) with the given exposure taken from the preceding study visit (e.g., lagged). A similar exploratory analysis was also conducted to investigate the association between fat-free mass (using the FFMI) and waist-hip ratio (exposures) and total BA levels (outcome). Models were adjusted for time since baseline visit, age at baseline, menarchal status [pre- (ref.) or post-], race [White (ref.) *vs*. non-White], and breast morphological stage [A, B, C, D, D/E, or E (ref.)]. Benjamini–Hochberg false discovery rate (FDR) adjusted *P*-values were calculated to account for multiple testing.

## Results

Eighty-two participants (aged 10.9 ± 1.4 SD years at baseline; **Table 1**) were included in the analyses. Participants contributed an average of 2.59 blood samples over 1.20 years of follow-up (total = 215 samples). The majority were non-Hispanic White and of normal weight. At baseline, all were pre-menarchal, and most were mid-pubertal (59% Tanner III breasts). Average age at menarche was 12.46 years (data available in 62 subjects).

**Table 1:** Baseline characteristics of participants from the body weight and puberty study who contributed blood samples for bile acid analyses (*n* = 82).

Characteristic	NW *N* = 53	OW/OB *N* = 29	Overall *N* = 82
Age (years; mean, SD) *^1^*	11.3 (1.3)	10.1 (1.2)	10.9 (1.4)
Race and ethnicity (n, %)*^2^*			
Black Or African American	13 (24.5%)	11 (37.9%)	24 (29.3%)
Hispanic	2 (3.8%)	7 (24.1%)	9 (11.0%)
Non-Hispanic White	35 (66.0%)	10 (34.5%)	45 (54.9%)
Other	3 (5.7%)	1 (3.4%)	4 (4.9%)
BMI (kg/m^2^)*^3^*	17.7 (16.4, 18.7)	23.7 (22.0, 25.5)	18.8 (17.0, 22.1)
BMI z-score*^1^*	−0.1 (0.7)	1.7 (0.4)	0.5 (1.1)
Percent total body fat*^1^*	26.7 (6.5)	41.4 (4.7)	31.9 (9.2)
Fat-free mass index (kg/m^2^) *^1^*	11.7 (1.1)	12.9 (1.3)	12.1 (1.3)
Waist:hip ratio*^3^*	0.80 (0.78, 0.84)	0.89 (0.86, 0.92)	0.84 (0.79, 0.88)
Breast Tanner stage (*n*, %)*^2^*			
I	1 (1.9%)	5 (17.2%)	6 (7.3%)
II	0 (0.0%)	2 (6.9%)	2 (2.4%)
III	36 (67.9%)	12 (41.4%)	48 (58.5%)
IV	8 (15.1%)	4 (13.8%)	12 (14.6%)
V	8 (15.1%)	6 (20.7%)	14 (17.1%)
Pubic hair Tanner stage (*n*, %)*^2^*			
I	6 (11.3%)	6 (20.7%)	12 (14.6%)
II	7 (13.2%)	2 (6.9%)	9 (11.0%)
III	19 (35.8%)	7 (24.1%)	26 (31.7%)
IV	16 (30.2%)	9 (31.0%)	25 (30.5%)
V	5 (9.4%)	5 (17.2%)	10 (12.2%)
Breast morphological stage (*n*, %)*^2^*			
A	1 (1.9%)	8 (27.6%)	9 (11.0%)
B	2 (3.8%)	4 (13.8%)	6 (7.3%)
C	10 (18.9%)	5 (17.2%)	15 (18.3%)
D	15 (28.3%)	8 (27.6%)	23 (28.0%)
D/E	18 (34.0%)	1 (3.4%)	19 (23.2%)
E	7 (13.2%)	3 (10.3%)	10 (12.2%)

*^1^*Mean (SD); *^2^n* (%); *^3^*Median (Q1, Q3).

NW, normal weight; OW/OB, overweight or obese; SD, standard deviation, Q1, first quartile, Q3, third quartile.

**Figure 2:**
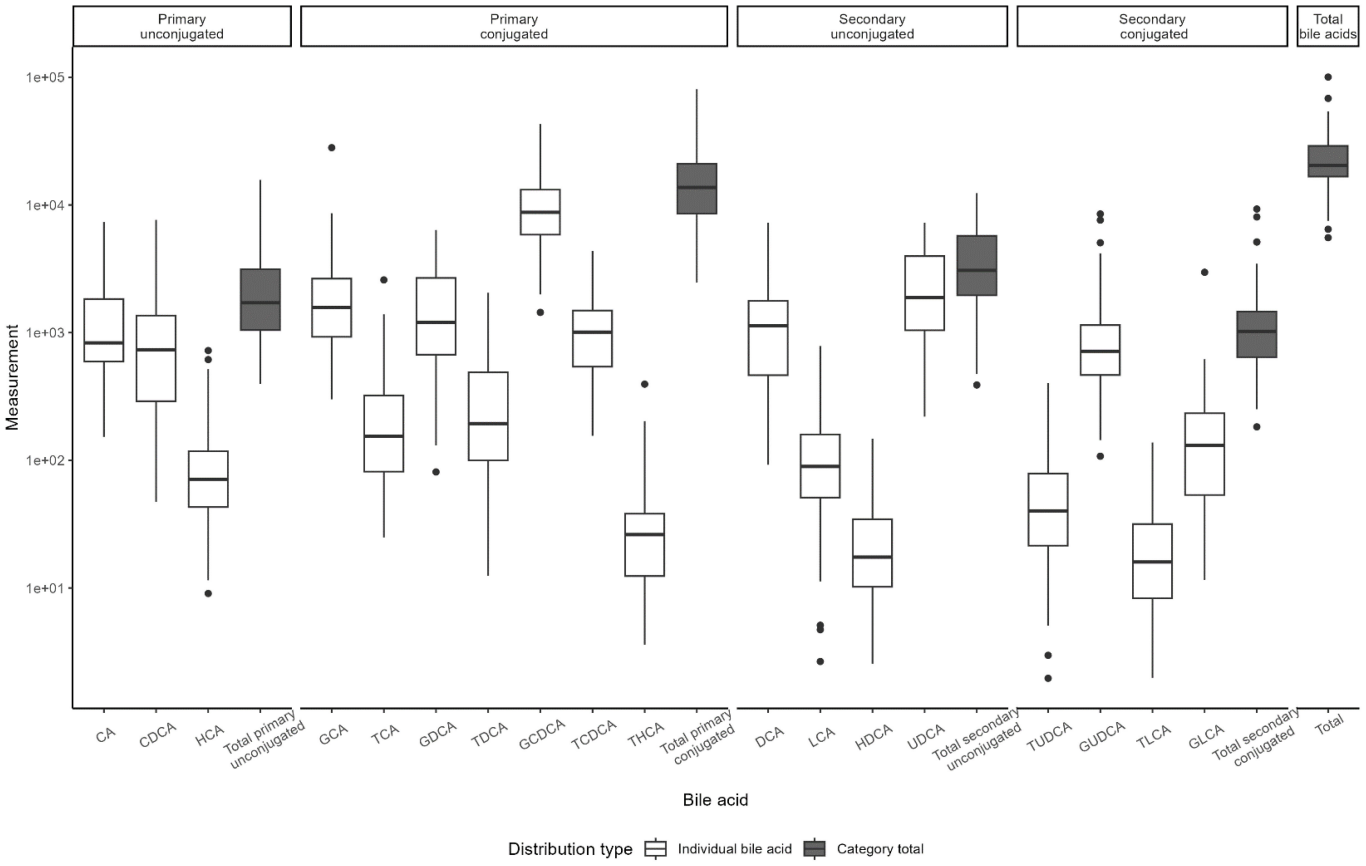
Distribution of bile acid levels by type. Boxplots of bile acid distribution as measured in Visit 1 samples. Boxes represent the interquartile range (IQR), with the line indicating the median. Whiskers extend to 1.5*IQR width from the box; data outside this range are outliers.

**Figure 3:**
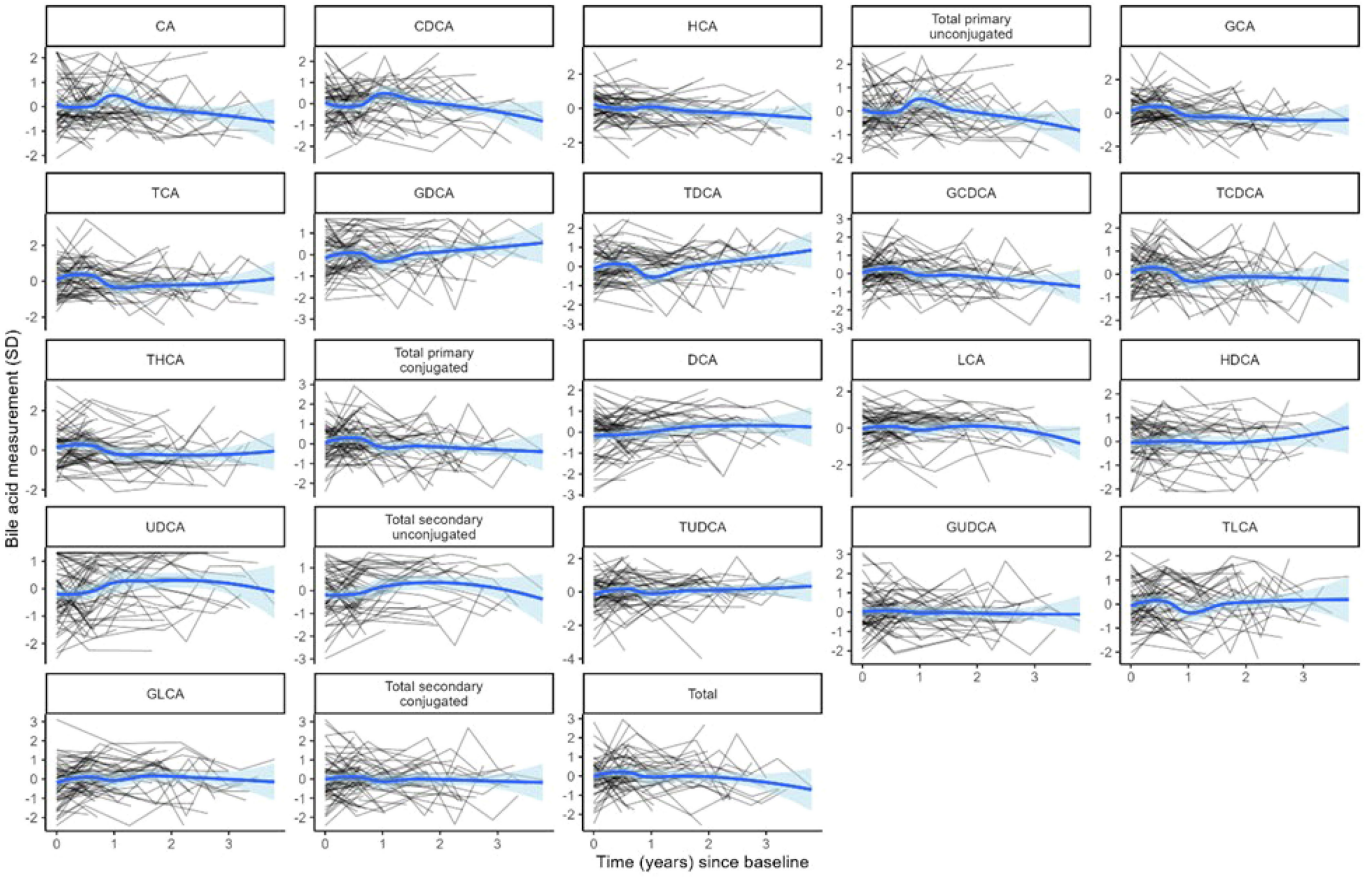
Log-transformed and standardized bile acid levels over time. Each black line represents the trajectory of a participant’s bile acid. The blue line represents the average of each bile acid over time in the cohort. Blue shading indicates the 95% confidence interval for the cohort average.

BA distributions from participants’ first samples are shown in **Figure 2**. Consistent with previous studies in non-fasting adolescents (13, 24), the BA pool was dominated by glycine-conjugated BAs, with glycochenodeoxycholic acid (GCDCA) representing 40.1% of total BA species. Primary BAs made up approximately three-fourths of the BA pool. Of note, hyocholic acid (HCA) has recently garnered attention as a novel biomarker of metabolic syndrome (25) and has not been measured in other pediatric cohorts. We observed very low levels of HCA and hyodeoxycholic acid (HDCA, an HCA species) in adolescent girls (<1% BA pool), consistent with studies in adults (25). BA levels were stable over time (**Figure 3**). BAs and composition did not change following the attainment of menarche (total BAs: p_FDR_ = 0.85; individual BAs p_FDR_: 0.33–1.0), and total BAs were not related to breast morphological stage (Type III joint test p_nominal_ = 0.11).

**Figure 4:**
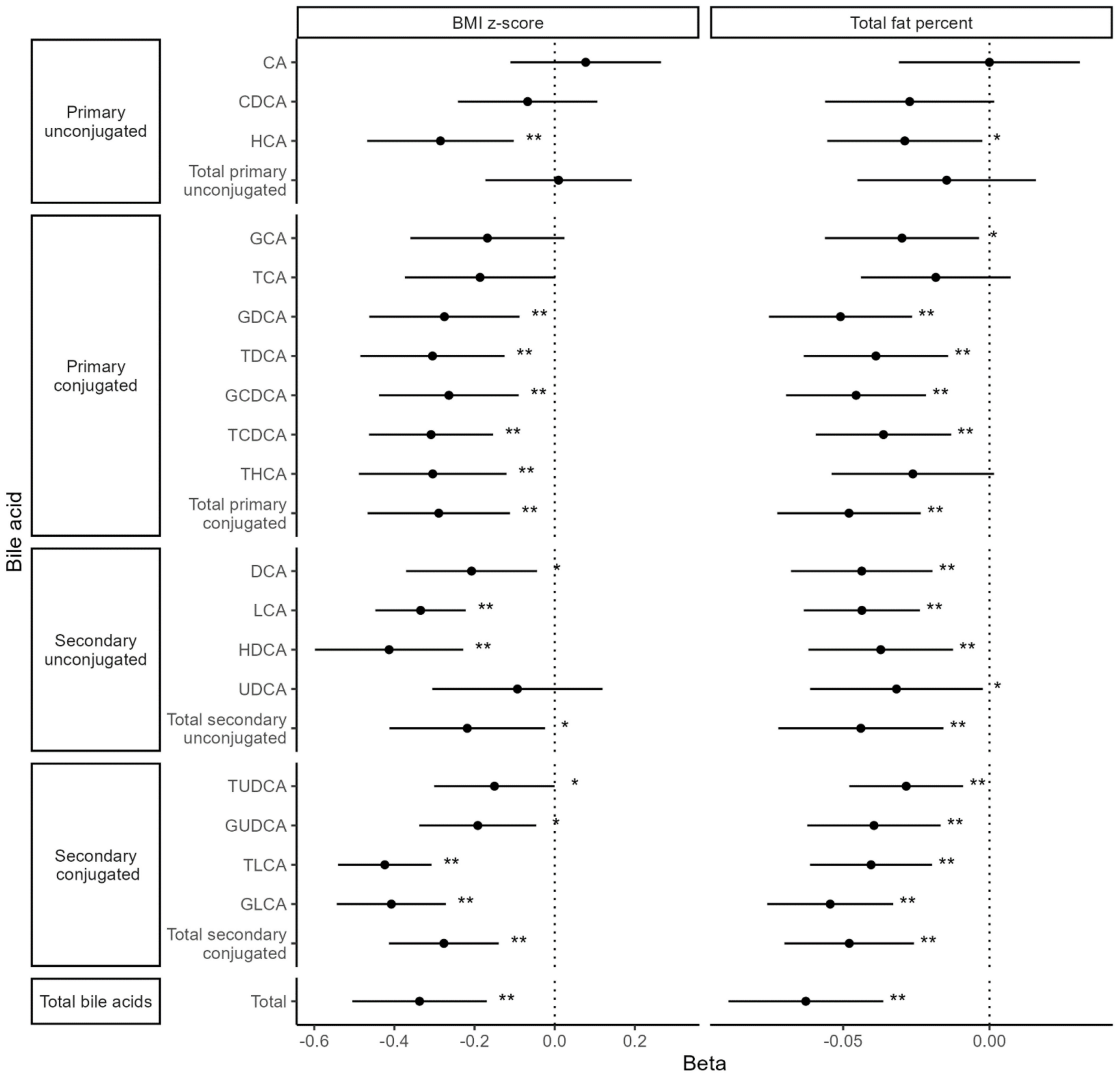
Associations between body composition metrics and bile acid levels. Values represent the estimated mean change in bile acid z-score (outcome) for a 1 unit increase in body composition variable (exposure). Models were adjusted for time since baseline visit, age at baseline, menarche status, race (White vs. non-White), and breast morphological stage. **Statistical significance after multiple testing correction, *Nominal significance.

**Figure 5:**
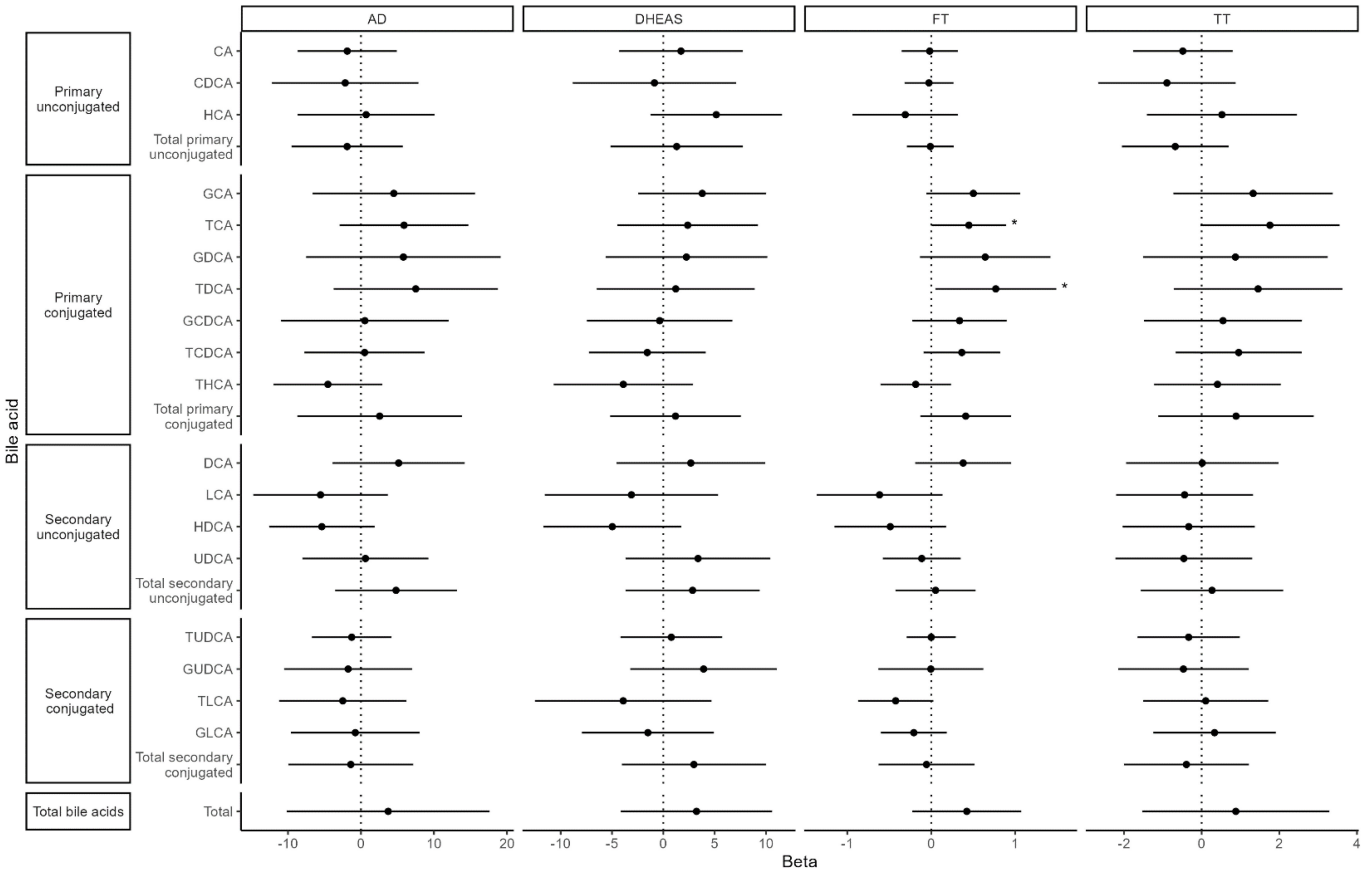
Associations between bile acids and hormones. Values represent the estimated mean change in androgen hormone z-score (outcome) for a 1 SD increase in bile acid z-score (exposure). Models were adjusted for time since baseline visit, age at baseline, menarche status, race (White vs. non-White), and breast morphological stage.

Both BMI and TBF were significantly negatively associated with total BAs [BMI: β = −0.34 (95% confidence interval (CI): −0.51, −0.17), p_FDR_ = 0.001; TBF: β = −0.06 (95% CI: −0.09, −0.04), p_FDR_ = 0.0001] as well as with 11 of 18 individual BA species (**Figure 4**). Fat-free mass index and waist-hip ratio were not associated with total BA levels [fat-free mass index: β = −0.11 (−0.30, 0.08), p_nominal_ = 0.25; waist-hip ratio: β = −0.007 (−0.014, 0.001), p_nominal_ = 0.07]. FT was nominally positively associated with two primary, conjugated BAs: taurocholic acid [TCA; β = 0.45 (95% CI: 0.01, 0.89), *p* = 0.047] and taurodeoxycholic acid [TDCA; β = 0.77 (95% CI: 0.05, 1.49), *p* = 0.036]. There were no associations between TT, AD, DHEAS, and BAs (**Figure 5**).

## Discussion

While traditionally thought to primarily aid in lipid digestion, BAs are now recognized as key signaling molecules that regulate inflammation, glucose and energy metabolism, appetite (26, 27), and perhaps even puberty (24). We were motivated to investigate the relationship between BAs, body composition, and androgens in children based on a growing body of literature linking BAs to metabolism in adults (28) and to PCOS (18). In the current studies, we demonstrate that in healthy pubertal girls there is an inverse relationship between body fat and BAs, as determined by DXA. Our studies also suggest a positive association between BAs and testosterone but not with adrenal androgens.

Some (25, 29, 30), but not all (31–33), studies in adults have also demonstrated an inverse relationship between BMI or body fat indicators and BAs either in the fasting state or after a liquid meal. The corresponding literature in children is more limited. A cross-sectional study by Higgins et al. of 30 Canadian children with obesity and 15 children of normal weight (mean age: 15 years) also demonstrated that most postprandial BAs were negatively correlated with BMI z-score and body fat percentage; however, BAs were measured using immunoassay with only a small subset also profiled by LCMS (*n* = 6 per group). A larger study of healthy Chinese children (12) reported that several BAs were lower in children with obesity than in normal-weight controls, whereas a study of Italian youth did not report a difference (11). Lastly, two metabolomic studies using fasting serum samples identified a negative association between a BA (HDCA or taurolithocholate 3-sulfate) and BMI in children (34, 35), whereas a third study using fecal samples reported a positive association (36).

The mechanism underlying the observed inverse relationship between body fat and BAs in girls is unclear but may involve insulin resistance. Of note, we did not measure insulin in the current studies, but adiposity is known to be the strongest predictor of insulin resistance in children (37). Two recent studies in children (13) and adults (25) demonstrated that greater insulin resistance or higher insulin levels after an oral glucose load were associated with lower levels of BAs. Hyperinsulinemic-euglycemic clamp studies demonstrated that insulin acutely decreases serum BAs in healthy adults of normal weight (33), and studies in mouse hepatocytes and HepG2 cells found that hyperinsulinemia suppressed hepatic transcription of CYP7A1 (38). It is interesting to speculate that insulin induction of BA production may be blocked selectively as an adaptive response to obesity. For example, BAs have an anorectic effect via action at hypothalamic TGR5 receptors (26, 27) and promote GLP-1 (39) and peptide YY (40) secretion. BAs also act on FXR and TGR5 receptors in white adipocytes to modulate adipocyte differentiation, lipid accumulation, adipokine and insulin signaling, and inflammation (41) and induce energy expenditure via activation of the TGR5/adenylate cyclase/deiodinase type 2 pathway in brown adipose tissue and skeletal muscle (42).

An alternative explanation for the inverse relationship between body fat and BAs in the current studies involves differences in the microbiome. The gut microbiome plays an important role in BA metabolism (i.e., deconjugation, dehydroxylation, and oxidation) and has been shown to be affected by body weight/composition (43). Adults with obesity have been shown to have both gut dysbiosis and changes in the BA pool composition, leading to the initiation of clinical trials to test microbiome-based treatments (e.g., fecal microbiota transplantation) and BA-based treatments (e.g., FXR agonists) to manage obesity and related conditions [reviewed in (44)].

We also observed a nominally significant positive association between FT and two primary, conjugated BAs. To date, the association between androgens and BAs has only been investigated in one specific population—Chinese women with PCOS—with mixed results. For example, Zhang et al., 2019 and Zhu et al., 2024 both reported that women with PCOS had higher concentrations of primary BAs than controls as well as a positive correlation between a subset of BAs and TT (16, 17). In line with these findings, a third study (14) found that women with PCOS with hyperandrogenism had higher fasting levels of primary BAs than women with PCOS without hyperandrogenism. However, two other studies reported lower BAs in women with PCOS compared with controls in stool and serum (18) and no correlation between serum TT and follicular fluid BA levels in women with PCOS (45). The connection between BAs and androgens remains unclear; however, a recent translational study suggests that in PCOS, alterations in the gut microbiota and BA profile may play a role in ovarian dysfunction: transplantation of fecal microbiota from women with PCOS into mice altered BA metabolism, disrupted estrous cycles, and caused infertility through a pathway involving interleukin-22 and small intestinal immune cells (18). Thus, it is possible that in healthy girls, as in women with PCOS, androgens may modulate BAs by influencing the landscape of the gut microbiome, but this hypothesis requires further study.

Most BAs were relatively stable during our longitudinal study. Of note, we did not identify changes in BA composition relative to menarche. This finding contrasts with that of Vanden Brink et al., who observed changes in the BA composition (e.g., ratio of conjugated to unconjugated species) in 10 healthy girls (average age: 7 years) presumed to be pre-pubertal or early pubertal compared with 10 post-menarchal girls (average age: 12 years). A similar pattern was observed by the authors in BAs during pubertal maturation in female rats (24). Importantly, however, these clinical studies were cross-sectional in nature, pubertal status was unknown in the younger girls, and samples from the younger girls were collected in the fasting state, whereas samples from post-menarchal girls were non-fasting.

Our study was strengthened by its longitudinal design with multiple samples (2–3, on average) collected from each participant. We also utilized gold standard techniques, including mass spectrometric analysis of serum BAs and androgens and determination of body composition via DXA. However, the sample size was relatively small, BAs were measured in non-fasting samples, no dietary information was collected, and the stool microbiome, insulin sensitivity (or surrogate markers, such as adiponectin), serum amino acids, and liver function were not assessed. Hormone levels may have also been influenced by menstrual cycle phase in a subset of samples. In contrast to our previous exploratory study that utilized untargeted metabolomics in this cohort and reported nominal, positive associations between three BAs and serum AD (9), we only found a nominally positive association between BAs (total and most BA species) and FT. The cause of these discrepant results is unclear but may reflect the significant differences in methodology and/or that the association between BAs and AD in the untargeted metabolomics investigation was only nominally significant.

In summary, alterations in BA profiles have been reported among adults with obesity, insulin resistance, and PCOS. The current studies reinforce our previous finding that in healthy pubertal girls, there is a negative association between BMI (or TBF) and BAs across a spectrum of body weights. These analyses did not replicate the nominal, positive associations we observed between BAs and AD in untargeted metabolomics analyses. We also observed a positive association between two BAs and FT that warrants further exploration, with particular attention to differences in the gut microbiome. Together, these results suggest potential biological links between altered BA signaling, overweight/obesity, and androgen production among pubertal girls.

## Data Availability

The original contributions presented in the study are included in the article/supplementary material. Further inquiries can be directed to the corresponding author.
